# Ancient conservation of androglobin expression reveals its evolutionary link to ciliary processes

**DOI:** 10.1093/molbev/msag147

**Published:** 2026-06-12

**Authors:** Carina Osterhof, David Teschner, Michelle Balling, Antonia Herwig, Charlotte Duda, Gaëlle Botton-Amiot, Andreas Hildebrandt, Simon Sprecher, Thomas Hankeln, David Hoogewijs

**Affiliations:** Institute of Organismic and Molecular Evolution, Molecular Genetics and Genome Analysis, Johannes Gutenberg University, Mainz, Germany; Department of Endocrinology, Metabolism and Cardiovascular System, University of Fribourg, Fribourg, Switzerland; Institute of Computer Science, Johannes Gutenberg University, Mainz, Germany; Institute of Organismic and Molecular Evolution, Molecular Genetics and Genome Analysis, Johannes Gutenberg University, Mainz, Germany; Department of Endocrinology, Metabolism and Cardiovascular System, University of Fribourg, Fribourg, Switzerland; Department of Endocrinology, Metabolism and Cardiovascular System, University of Fribourg, Fribourg, Switzerland; Department of Biology, Institute of Zoology, University of Fribourg, Fribourg, Switzerland; Institute of Computer Science, Johannes Gutenberg University, Mainz, Germany; Department of Biology, Institute of Zoology, University of Fribourg, Fribourg, Switzerland; Institute of Organismic and Molecular Evolution, Molecular Genetics and Genome Analysis, Johannes Gutenberg University, Mainz, Germany; Department of Endocrinology, Metabolism and Cardiovascular System, University of Fribourg, Fribourg, Switzerland

**Keywords:** androglobin, oxygen biology, globin evolution, ciliary function, functional divergence, multicellularity

## Abstract

Androglobin (Adgb) is the most distinctive member of the globin superfamily. Its characteristic globin domain is permuted, interrupted by a calmodulin-binding motif, and embedded within a large multidomain protein of ∼1500 amino acids that also contains a calpain protease domain. Initially described as testis-specific in mammals, Adgb is also expressed in ciliated epithelia of the female reproductive tract, lung, and brain, and knockout studies reveal its pivotal role during spermatogenesis. To trace its evolutionary origin, we performed comprehensive phylogenetic analysis across diverse eukaryotic taxa. Adgb is present in all major flagellated eukaryotic lineages but absent from nonflagellated clades. Orthology analysis indicates Adgb has been maintained as a predominantly single-copy gene across >1 billion years of evolution—a pattern contrasting sharply with other globins that underwent repeated duplication and functional diversification. Analysis of publicly available transcriptomes from early-branching metazoans confirmed robust Adgb expression in ciliated cell types and provides evidence for its regulation by the ancient ciliogenic transcription factor cRFXa in the choanoflagellate *Salpingoeca rosetta*. RNA in situ hybridization validated these findings, and comparative analyses suggest that ancestral Adgb homologs lacked the permuted globin domain found in metazoans. Collectively, our results demonstrate that Adgb exemplifies a rare evolutionary trajectory where structural innovation (domain permutation and fusion) enabled functional specialization while being an integral part of the nonredundant ciliary machinery. Adgb thus illustrates how constraint and innovation combine to shape the long-term fate of a protein.

Significance Globins are ancient proteins with diverse functions, but most lineages have undergone repeated duplication, loss, and functional turnover. Here we demonstrate that androglobin (Adgb), an unusual multidomain globin, is exceptionally conserved across flagellated eukaryotes and tightly coupled to ciliary cell types. Our findings reveal how a rare structural innovation transformed Adgb into an indispensable regulator of ciliary function, illustrating how both evolutionary constraint and innovation shape the long-term fate of gene families.

## Introduction

Globins are a family of small metalloproteins, which share a characteristic α-helical protein structure and an iron atom-containing heme prosthetic group (Kendrew et al. 1958; Dickerson and Geis 1983; Bolognesi et al. 1997). The globin fold is widely distributed over the tree of life, with occurrences in Archaea, Bacteria, and Eukaryotes (Vinogradov et al. 2005, 2006). It is hypothesized that the fold structure evolved only once and gave rise to many different globin lineages with a wide variety of functions including oxygen transport, storage and sensing, nitric oxide and reactive oxygen species metabolism, redox sensing, and enzymatic function (Vinogradov and Moens 2008; Keppner et al. 2020).

Historically, the globin protein family served as a model to study different forms of evolutionary radiation. The high structural conservation coupled with several duplication events has rendered the globin protein family an excellent case study for fundamental principles of molecular evolution. Lineage-specific gene radiation events in specific phylogenetic classes, such as those observed in Rhabditidae and Drosophilidae enabled us to study the principles of sub- and neofunctionalization (Burmester et al. 2006; Hoogewijs et al. 2007; Prothmann et al. 2020). The synteny signatures of paralogous hemoglobin, myoglobin, and cytoglobin allowed us to trace the whole-genome duplication events in early vertebrate evolution. The developmental switch between embryonic, fetal, and adult hemoglobins is a sophisticated example of how gene family expansion can be coupled with regulatory evolution to meet changing physiological demands (Storz 2016; Hardison 2025). Globins are expressed in many different tissues and cell types where they may fulfill additional, nonclassical cellular functions beyond oxygen transport and storage, such as the protection against reactive oxygen or nitrogen species, the prevention of hypoxia-triggered apoptosis, or the involvement in intracellular signaling processes (Burmester and Hankeln 2014; Keppner et al. 2020).

In 2012, androglobin (Adgb) was identified as the newest and—at the same time—most peculiar member of the vertebrate globin protein family (Hoogewijs et al. 2012). Adgb is a large chimeric protein of about 1500 amino acids, which contains an embedded globin domain. This globin domain is permuted with respect to its characteristic alpha helices, and it is interrupted by a calmodulin binding motif.

Adgb was initially identified bioinformatically in the echinoderm species *Strongylocentrotus purpuratus* and the cephalochordate *Branchiostoma floridae*. Subsequent phylogenetic analysis revealed that Adgb homologs exist in nearly every branch of the metazoan tree. Orthologous copies of the Adgb gene could be found in basal metazoan taxa such as the cnidarian *Nematostella vectensis* (Cnidaria), the placozoan *Trichoplax adhaerens* (Placozoa) and even in the choanoflagellate *Monosiga brevicollis* (Hoogewijs et al. 2012), which suggests an elementary and possibly conserved function for Adgb in animals. In phylogenetic studies, the Adgb globin domain consistently clusters as a well-supported monophyletic branch at the base of the metazoan globin sequence tree (Hoogewijs et al. 2012), indicating an early origin and underscoring Adgb as a deeply conserved functionally and evolutionary distinctive member of the globin family.

The gene name hints at the predominant expression in mammalian testis tissue, where lower levels of Adgb could be correlated with impaired fertility (Hoogewijs et al. 2012). More recently, it was shown that Adgb is expressed in multiciliated cells of the mammalian lung, brain, and the female reproductive tract (Koay et al. 2021). Upon ablation of this flagellar gene, severe phenotypes can be observed: A study in Adgb-deficient mice confirmed its pivotal role in reproduction, demonstrating that Adgb ablation leads to fertility impairments and structural changes in sperm morphology (Keppner et al. 2022). These findings were later confirmed in human patients (Qu et al. 2023; Gao et al. 2025). The exact mechanism, however, by which Adgb impacts flagellar motility remains unknown. Fertility impairments greatly aggravate breeding of the Adgb knockout line and complicate functional studies. Additionally, studying motile ciliary defects in lungs or other tissues poses particular challenges in mammals, since beat frequencies need to be monitored live (Jing et al. 2017) or in sophisticated cell culture systems (Lee et al. 2020). Due to Adgb gene loss events in the evolution of *Drosophila melanogaster* and *Caenorhabditis elegans*, these two canonical nonmammalian model systems are not suitable for Adgb functional analysis (Hoogewijs et al. 2012). The latter is particularly striking as the *C. elegans* globin gene repertoire contains more than 30 globin genes (Hoogewijs et al. 2008). Both spermatogenesis and ciliogenesis are strongly conserved processes (Mitchell 2007), and recent reports suggest that the Adgb lineage could be much older than originally described (Joachimiak et al. 2021).

Fueled by the lack of canonical model systems for functional studies, we examined the phylogeny of Adgb in unicellular eukaryotes as well as the expression pattern of Adgb in taxa at the root of the metazoan tree and analyzed whether the gene structure and distinct expression of Adgb are conserved. We detected conserved Adgb expression, regulated by an ancient ciliogenesis transcription factor and experimentally validated these findings via RNA scope staining of Adgb mRNA in basally branching metazoan species. Unexpectedly, modeling data indicated that Adgb from basal, unicellular species lacks the characteristic permuted globin domain. Altogether, our results demonstrate that not only the Adgb gene but also its mRNA expression pattern is highly conserved, confirming its evolutionary ancient origin and its presence in the basal ciliary gene repertoire. Importantly, we identified model organisms that could facilitate to further unravel Adgb's molecular role in the development or maintenance of metazoan ciliary and flagellar structures.

## Results

### Adgb homologs are widely distributed across flagellated eukaryotic lineages

**Figure 1:**
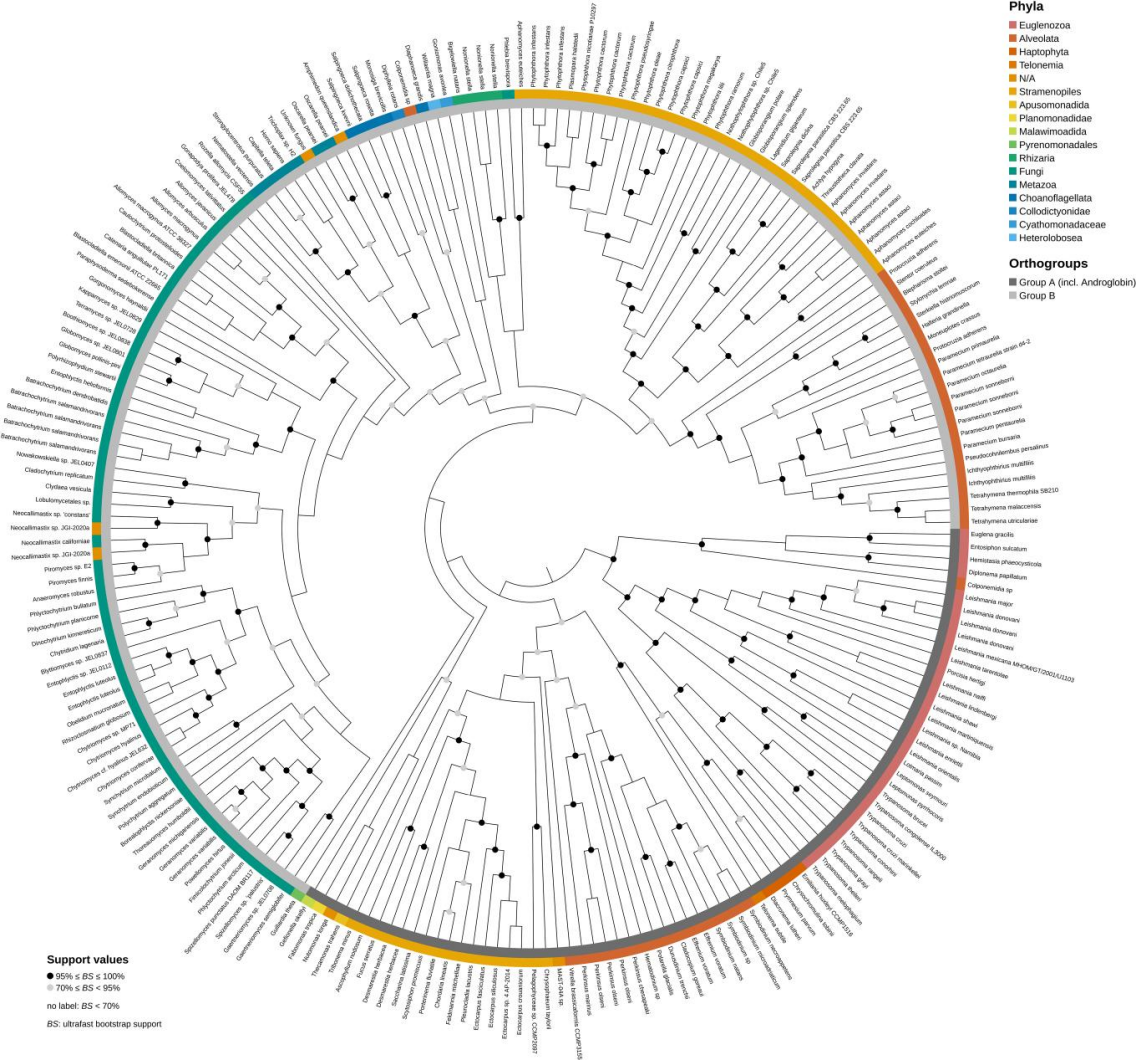
Maximum likelihood reconstitution of Adgb phylogeny across eukaryotes. Affiliation with the different phyla is indicated by the color in the outer circle. Bootstrap support is depicted as circle on all nodes. Bootstrap between 100% and 95%: black circle; bootstrap between 95% and 70%: gray circle; no circle: support lower than 70%. The circle “Orthogroups” refers to the clustering of sequences performed by the possvm algorithm, which predicts two paralogous groups of Adgb sequences distributed over a wide variety of taxa.

Adgb was initially described as metazoan-specific (Hoogewijs et al. 2012), but a putative homolog with ciliary association was recently identified in the ciliate *Tetrahymena thermophila* (Joachimiak et al. 2021), suggesting a broader evolutionary distribution. Since flagellar structures were probably already present in the last eukaryotic common ancestor, we expanded our search for Adgb homologs to other unicellular, eukaryotic lineages. Through reciprocal BLAST searches using three phylogenetically diverse Adgb seed sequences (human, sponge, *Tetrahymena*), we identified 220 putative Adgb homologs. Candidate sequences were retained if they met a minimum length threshold (>800 aa) and, upon reverse BLAST against the proteome of the seed sequence species, returned at least one Adgb sequence among the top hits. This relaxed reciprocity criterion was chosen to account for the broad evolutionary distances involved. A multiple sequence alignment demonstrated significant sequence divergence across the lineages, and only very few conserved positions remained. The Maximum likelihood phylogenetic tree derived from this alignment can be found in Fig. 1.

Vertebrate Adgb is a multidomain protein, with a calpain-like domain, globin domain, an IQ motif and a long coiled-coil domain at the C terminus (Hoogewijs et al. 2012). To assess domain composition in the new Adgb sequences, we annotated the sequences using InterProScan with searches against the CDD, PFAM, PANTHER, InterPro, and SUPERFAMILY databases. Ninety-eight percent of sequences were classified as “Androglobin” by the PANTHER database (PTHR46298), 98% belong to the SUPERFAMILY Cysteine proteinases (SSF54001), and 90% of sequences were identified as “Calpain” (PF00648). The coiled-coil domain was found in 70% of the sequences and is predicted to be mostly absent in Euglenozoa. PFAM domains Androglobin II (PF22068, 23%), IV (PF22069, 33%), and V (PF22070, 13%) were found predominantly in Opisthokont sequences. Adgbs permuted globin domain and IQ motif could only be found in metazoan sequences (cd22307, 3%). A detailed overview of the domain composition per sequence is provided in File S1, and distribution over the phylogenetic clades is shown in Fig. S1.

The Adgb sequences cluster according to established eukaryotic phylogeny (Figs. 1 and  S1): metazoans and choanoflagellates are positioned as robustly supported sister to fungal sequences (Bootstrap support value: 93%), and additional monophyletic groups consisted of Euglenozoa (100%), Dinophyceae (99%), Oomycota (100%), and Ciliophora (100%). To distinguish true orthologs from paralogs, we applied the possvm algorithm to our tree, which reconciliates the gene tree with known species trees (Grau-Bové and Sebé-Pedrós 2021). This analysis partitioned the sequences into two groups. The Adgb sequences from Fungi, Choanoflagellata, Rhizaria, Oomycota, and Ciliophora are predicted to be truly homologous to human Adgb, whereas the other contained sequences are from Euglenozoa, Haptista, and Dinophyceae. However, phylogenetic support for this bipartition was weak (Bootstrap: 49%), and no extant lineage retained both sequence types. Overall, duplication of the Adgb gene is rare and only happens at the species level. Putative homologs from the same species are exclusively on neighboring branches, and these branches are usually of very short length (see eg Fig. S1, red box). It is therefore possible that these sequences rather represent isoforms or annotation artifacts than true functional paralogs.

The phylogenetic distribution of Adgb revealed a striking correlation with flagellar structures: Adgb is present in all major flagellated lineages (Opisthokonta, Haptophyta, Alveolata, Euglenozoa, and Rhizaria) but absent from nonflagellated clades (Amoebozoa, Centrohelida, higher Fungi, and Apicomplexa). Notably, within the Alveolata, Adgb is present in the flagellated Ciliophora, Dinophyceae, Perkinsozoa, and Colpodellida but is missing in the parasitic Apicomplexa, where flagella are absent in most life stages and biosynthesis and composition is greatly altered (Portman and Šlapeta 2014; Francia et al. 2016), demonstrating that ciliary coupling holds even at finer phylogenetic resolution. To test whether this molecular conservation extends to cellular expression patterns, we examined Adgb localization in multicellular organisms containing both ciliated and nonciliated cell types.

### Mammalian Adgb expression sites are mirrored in the cnidarians *N. vectensis* and *Hydra vulgaris*

**Figure 2:**
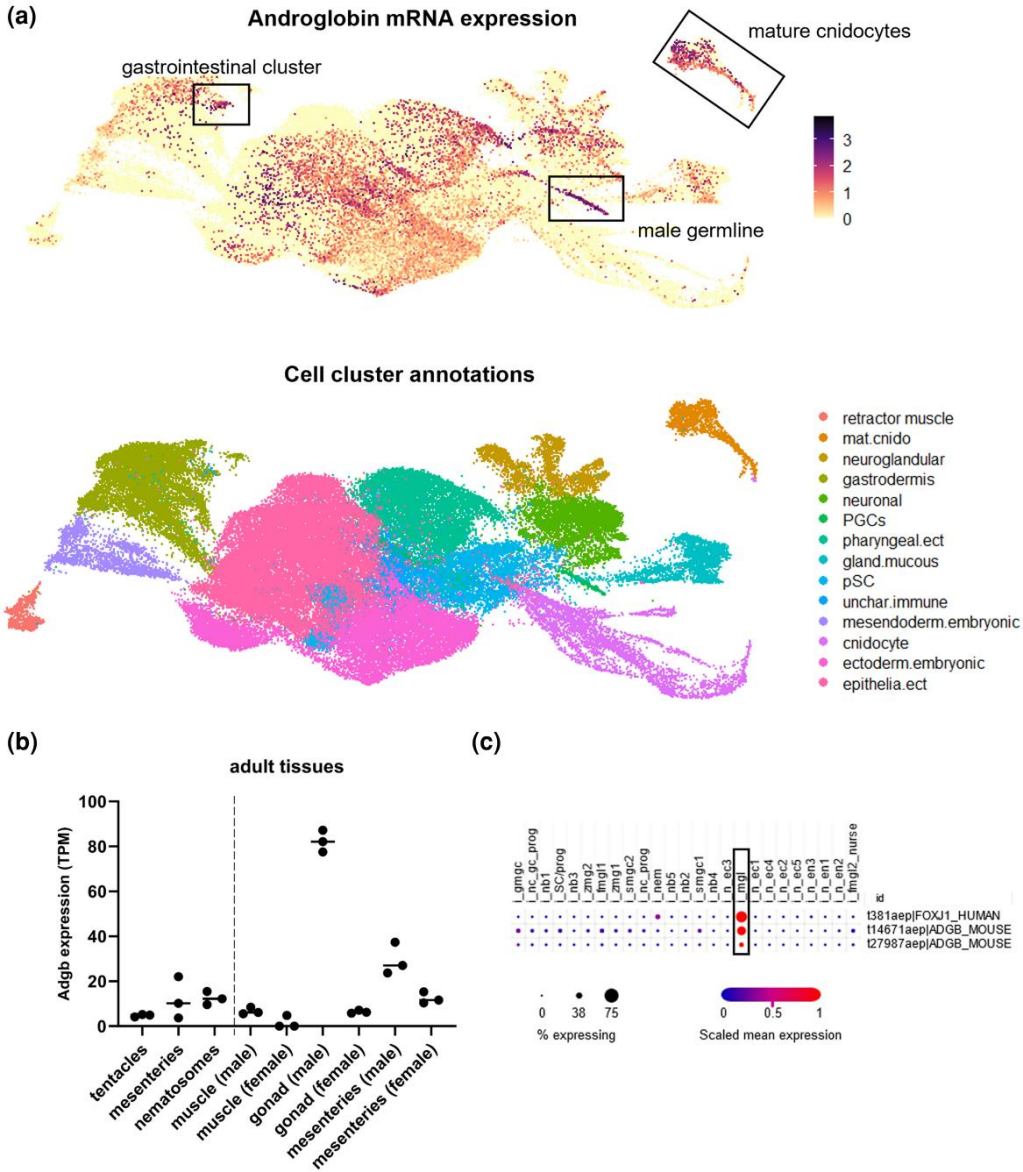
Adgb mRNA expression in different cnidarian model species. a) In scRNA-seq data derived from different developmental stages of *N. vectensis*, Adgb mRNA expression is highest in the male germline and in fully mature cnidocytes (data from Cole et al. 2024). b) Adgb mRNA expression in tissue-specific bulk RNA-seq data of *N. vectensis*. Expression is most abundant in male gonads, followed by male and female mesenteries. c) Heatmap displaying expression intensity of two Adgb transcript variants and Foxj1 in clusters of single cells from the interstitial lineage of *H. vulgaris*. Both Adgb transcript variants as well as Foxj1 are expressed strongest in the male germ line (mgl). Dataset: Siebert et al. 2019, image: single-cell atlas of Broad Institute, https://singlecell.broadinstitute.org/single_cell.

We first analyzed the Adgb expression in the two cnidarian model species, *N. vectensis* and *H. vulgaris*. Single-cell RNA-sequencing (scRNA-seq) analysis showed high levels of Adgb expression in the male germ line and mature cnidocytes from *Nematostella* (Fig. 2a). In line with this, most abundant Adgb mRNA expression was detected in bulk RNA-seq data from male gonads, followed by the mesenteries (Fig. 2b). The scRNA-seq data from *Hydra* also displayed the highest Adgb mRNA expression in male germ cells (Fig. 2c). In agreement with our mammalian data, it was strongly coexpressed with FoxJ1, a critical ciliogenesis transcription factor (Fig. 2c; Koay et al. 2021). The mesenterial expression in bulk RNA-seq data might partially be derived from residual germline tissue; however, single-cell data showed a positive subpopulation in the gastrodermal cluster that was distinct from the male germline (Fig. 2a). We performed gene ontology enrichment analysis on genes whose expression was positively correlated with Adgb's expression and found a clear ciliary signature in this cluster, indicating a second, ciliated cell type in *Nematostella's* gastrointestinal system (File S2).

**Figure 3:**
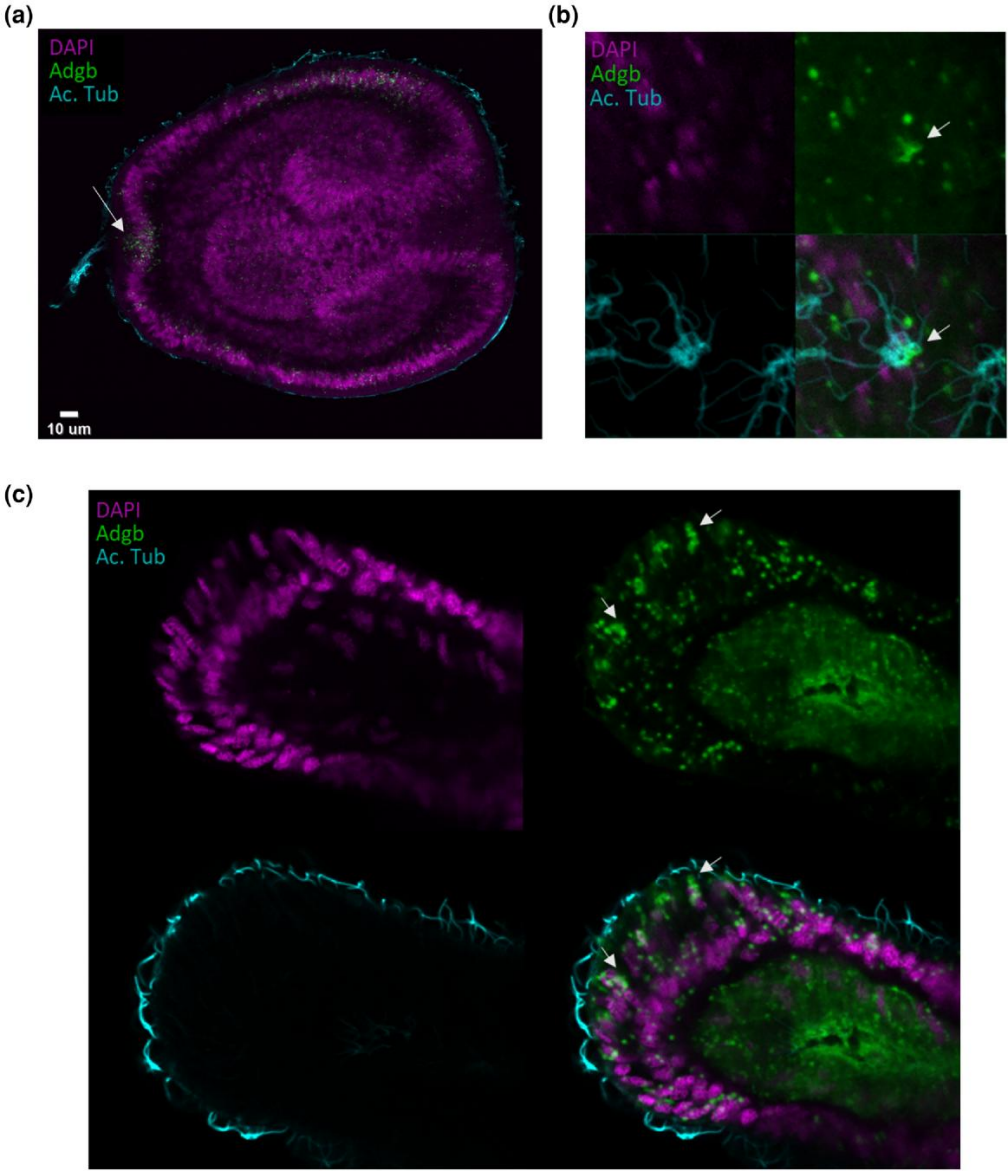
Adgb mRNA expression in different stages of *N. vectensis* development. a) In the planula stage, Adgb mRNA-positive cells are found throughout the epithelial layer, with an enrichment at the apical pole where the apical organ is formed. b) Multiciliated cells show stronger Adgb signal than surrounding cells. c) In tentacles of planula larvae, Adgb-positive cells are located near the surface and also carry multiple cilia.

In RNA-seq data derived from dissected early and late planula larvae of *Nematostella*, Adgb mRNA expression was more pronounced in tissue from the aboral site, where the ciliated organ is formed (Fig. S2). We confirmed this experimentally via RNA scope staining of Adgb mRNA in different developmental stages. Adgb mRNA expression was more pronounced at the apical end of the embryo (Fig. 3a). In later stages, high amounts of Adgb mRNA were detected in cells carrying multiple cilia (Fig. 3b) in the ciliated epithelia of larval tentacles (Fig. 3c).

### Ciliary and flagellar association of Adgb is confirmed in basal metazoans

**Figure 4:**
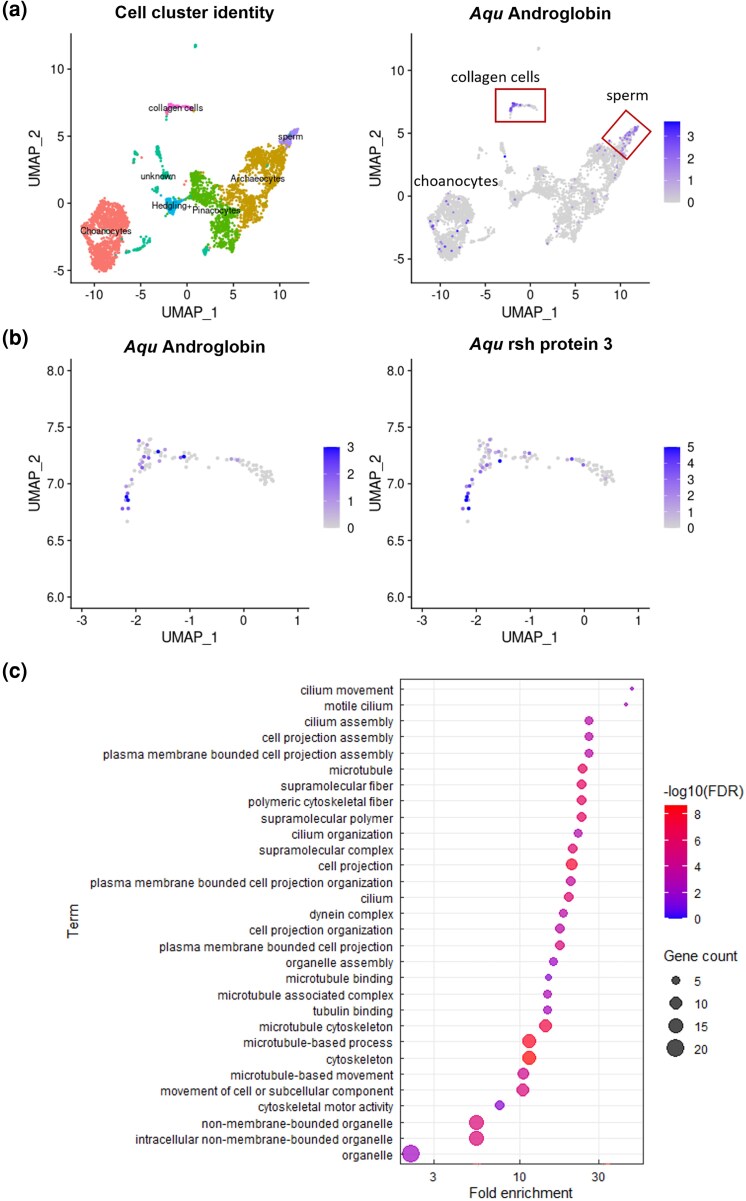
Adgb mRNA expression derived from scRNA-seq data of the sponge *A. queenslandica* (Aqu). a) Left: Uniform Manifold Approximation and Projection (UMAP) representation of cell types identified in Aqu. Right: Adgb mRNA expression is highest in sperm cells, a collagen-rich cell cluster, and in choanocytes. b) Enlarged representation of the “collagen cells.” Adgb expression follows the same gradient as the homolog of rsh3, an established ciliary component. c) Gene ontology analysis of marker genes highly expressed in the “collagen cells.” Enriched terms are strongly connected to motile cilia, indicating that the “collagen cells” are ciliated.

The strong conservation of Adgb expression sites in cnidarians prompted us to also consider more basal lineages in our analysis. Publicly available single-cell sequencing data from the poriferan *A. queenslandica* (Aqu) showed strong Adgb-positive signal in sperm cells and in a second cluster with high collagen levels (Fig. 4a). Adgb expression correlated with radial spoke head protein 3 (rsh3), a gene which is homologous to an important ciliary structural component (Fig. 4b). We performed gene ontology enrichment analysis on the marker genes of this second cluster and found a strong enrichment of cellular component terms related to motile cilia, such as “motile cilium” itself, “cilium movement,” and “cilium assembly,” indicating that the collagen cluster is ciliated (Fig. 4c; File S3). There was little to no Adgb mRNA expression in choanocytes, even though they carry a flagellum. However, a second dataset derived from sorted cell populations of Aqu showed significant amounts of Adgb mRNA in separate replicates of choanocytes derived from mainly one individual (Fig. S2a), hinting at interindividual expression differences or a role during distinct stages of choanocyte differentiation. A second, more detailed sponge dataset from Spongilla (Musser et al. 2021) could not be fully analyzed, since the 3′end crucial for proper quantification is missing. However, correlation analysis performed on the noisy Adgb mRNA expression data still suggests ciliary association (data not shown).

The association of Adgb and ciliated cells was also reflected during early development of *A. queenslandica*. Pseudotime-sorted transcriptomes of whole single embryos (Anavy et al. 2014) displayed an increase of Adgb expression during the motile larval phase, and a subsequent decrease when the larvae settle. Consistent with our cnidarian data, Adgb expression strongly correlated with the ciliary protein rsh3 (Fig. S3b).

**Figure 5:**
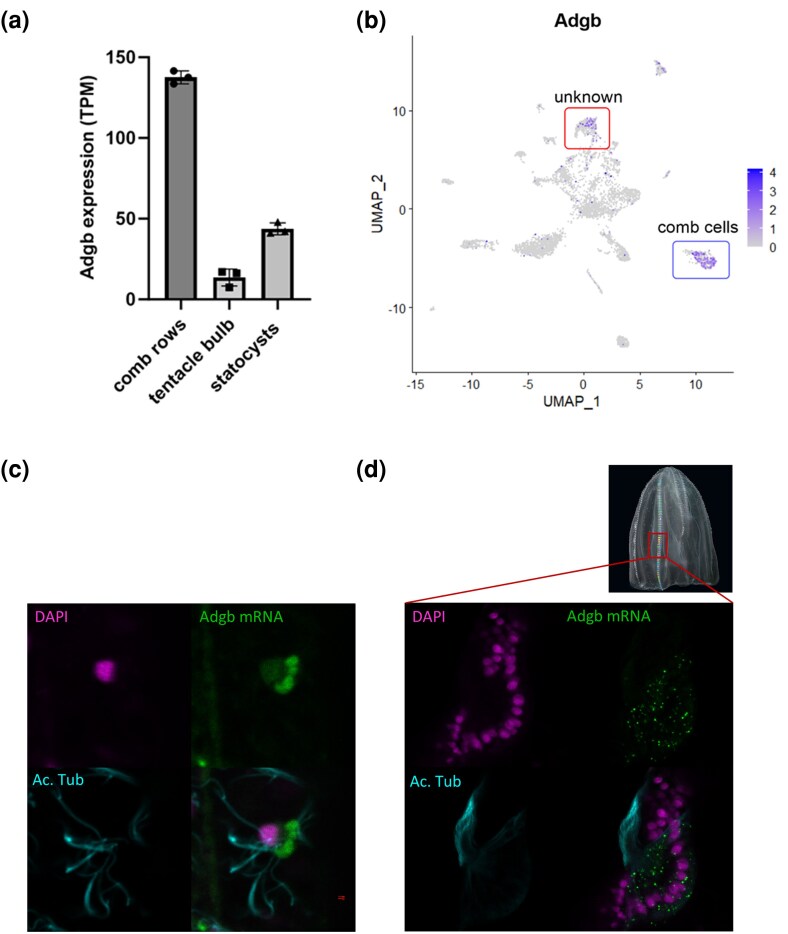
Adgb mRNA expression sites in the ctenophore *M. leidyi*. a) Bulk mRNA sequencing data from different isolated tissues show highest expression of Adgb in comb rows. b) scRNA-seq data show that comb row expression of Adgb is derived from ciliated comb cells. In addition, a second, unlabeled cluster displays Adgb-positive cells. c) Adgb mRNA detected in multiciliated cells via RNA Scope. d) Adult specimen of Mnemiopsis. On the surface, sail-like ciliary structures are found in rows (comb rows, red box). In the cross-section (multipanel figure), Adgb mRNA expression is found in the cellular structure from which the cilia forming the sail protrude. Magenta = DAPI (nuclei), green = Adgb mRNA; cyan = α-acetylated-tubulin antibody staining (cilia). Picture of Mnemiopsis: “Ctenophora *Mnemiopsis leidyi* 2” by Yuliia Baiandina/Wikimedia commons CC BY 4.0.

Next, we analyzed Adgb expression in the ctenophore model species *M. leidyi*. In bulk RNA-seq data derived from dissected tissues, Adgb expression was highest in the comb rows (Fig. 5a). ScRNA-seq analysis identified two Adgb-expressing clusters (Fig. 5b), the ciliated comb cells that drive expression in the comb rows and a second cluster that could not be annotated in the original analysis (“unknown,” Fig. 5b). Correspondingly, both clusters showed an enrichment for FoxJ1-positive cells (Figure S4). We identified marker genes significantly upregulated in the “unknown” cluster. These marker genes were enriched for gene ontology terms such as cilium, cilium movement and cilium organization, as well as terms connected to DNA replication and cell cycle (File S4). It is conceivable that these cells, which appear to be flagellated and mitotically active, represent the germ cells of *Mnemiopsis*. Via RNA Scope, we could experimentally confirm the bioinformatically inferred expression sites in *Mnemiopsis*. Adgb mRNA was enriched in multiciliated cells (Fig. 5c), very similar to *N. vectensis*. Moreover, Adgb mRNA was also strongly expressed in the ctenophore-specific, ciliated comb rows (Fig. 5d).

Finally, publicly available scRNA-seq data (Najle et al. 2023) suggested a strong correlation between Adgb mRNA expression and cilia-related genes in four different species of placozoa (Fig. S5; File S5), another basal metazoan lineage probably related to cnidaria (Laumer et al. 2018).

### Adgb is expressed in a choanoflagellate and regulated by a ciliary transcription factor

**Figure 6:**
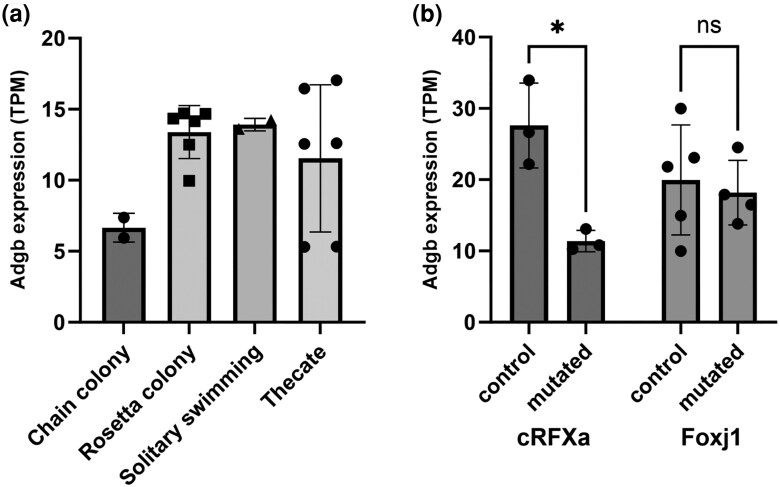
Adgb mRNA expression in the choanoflagellate *S. rosetta.* a) Adgb is expressed in solitary as well as colonial stages of *S. rosetta*, with a tendency of higher expression in solitary swimming and rosetta colony stages. Expression in thecate cells is strongly variable. b) Adgb mRNA expression is significantly reduced upon mutation of the ciliary transcription factor cRFXa, whereas ablation of Foxj1 has no effect on the regulation of Adgb expression.

Already upon discovery, a putative Adgb homolog was identified in the choanoflagellate species *M. brevicollis (*Hoogewijs et al. 2012*)*, a group that is considered to be the closest living unicellular relative of metazoans (King et al. 2008; Richter et al. 2018). Our analysis has identified a second choanoflagellate Adgb homolog in *Salpingoeca rosetta*, which is considered as a model for the emergence of multicellularity. Depending on environmental factors, *S. rosetta* exists in different forms: thecate, free swimming, and colony forming. RNA-seq data derived from different life stages (Fairclough et al. 2013) showed low to moderate Adgb expression in all forms, with high variability in thecate stages and lower values in chain colonies (Fig. 6a). Differential expression analysis in the original publication of the data even identified Adgb as enriched in the free-swimming cell population, a tendency we also observed upon data reevaluation, but less clearly pronounced. In a second dataset, expression of FoxJ1 and cRFXa was perturbed to analyze the effect of these transcription factors on ciliary gene expression (Coyle et al. 2023). Knockout of cRFXa in *S. rosetta* indeed resulted in a ciliary phenotype and significant downregulation of Adgb expression levels. Surprisingly, ablation of Foxj1 had no effect on ciliary movement or ciliary gene expression, including Adgb (Fig. 6b). Collectively, these data suggest that Adgb expression is associated with flagellated cells but precedes FoxJ1-mediated gene regulation.

### Ancestral Adgb lacks the permuted globin domain

**Figure 7:**
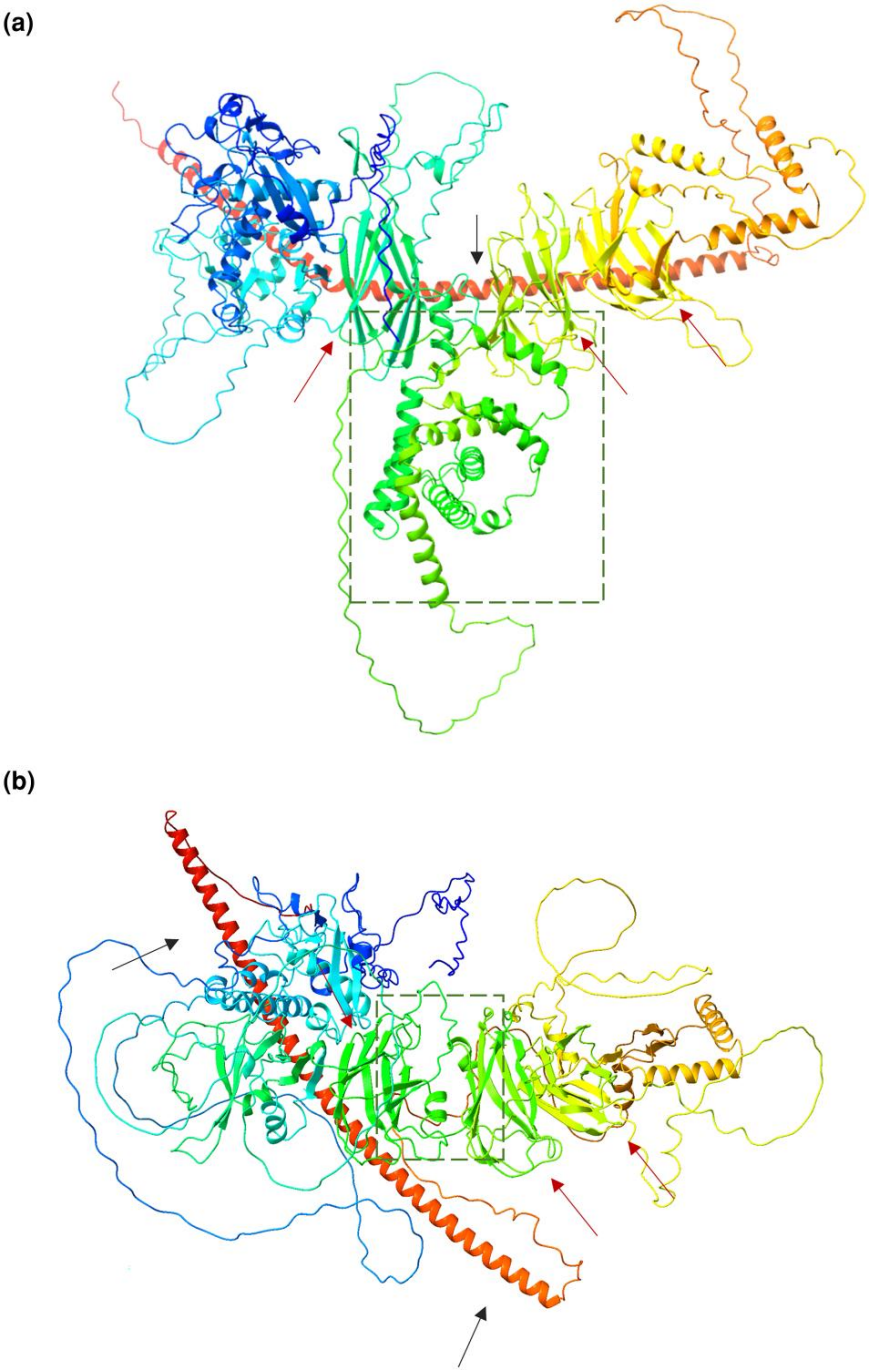
Prediction of Adgb structure from a) the demosponge *A. queenslandica* and b) the dinoflagellate *Symbiodinium necroappetens* by AlphaFold2. The overall topology of both proteins is similar, with three beta-sheet structures (red arrows) at the beginning and a long alpha helical tail at the C terminus (black arrows). In *Amphimedon*, the globin domain was found between beta-sheets 1 and 2 (a, dashed box). At the same position, *Symbodinium* only has a disordered, much shorter amino acid stretch (b, dashed box). Visualization was done with ChimeraX.

Since conservation of structural elements in proteins is often much higher than amino acid sequence similarity, we modeled the protein structure of our newly identified Adgb sequences (Fig. 1) with AlphaFold2, computing structural predictions for representative Adgb sequences of all major clades. All metazoan Adgb structures as well as the choanoflagellate proteins showed a similar overall topology: the calpain domain, consisting of several alpha helices, followed by three beta-sheet structures with the globin domain in the middle of the second (Fig. 7a). At the C terminus of the protein, a long helical extension was predicted. The structural order of an active center followed by three beta-sheets was similar to that of other calpains, e.g., the calpain 7 of the fish *Danio rerio* (Fig. S6, UniProt ID: B4F6P1). Intriguingly, no globin domain could be identified in the nonchoanocyte unicellular eukaryotes, where we found that the second barrel structure was also interrupted, but by a much shorter, unordered domain. No similarity to the helical globin domain was detectable. This finding is in line with the InterPro domain annotations (Fig. S1; File S1). Also here, no globin domains could be identified in nonmetazoan lineages. The C-terminal long helix, however, was structurally conserved in those Adgb orthologs (Fig. 7b). We therefore conclude that the ancestral Adgb was a calpain-like protein with a long C-terminal extension that acquired the permuted globin domain in the last common ancestor of choanoflagellates and metazoans.

## Discussion

**Figure 8:**
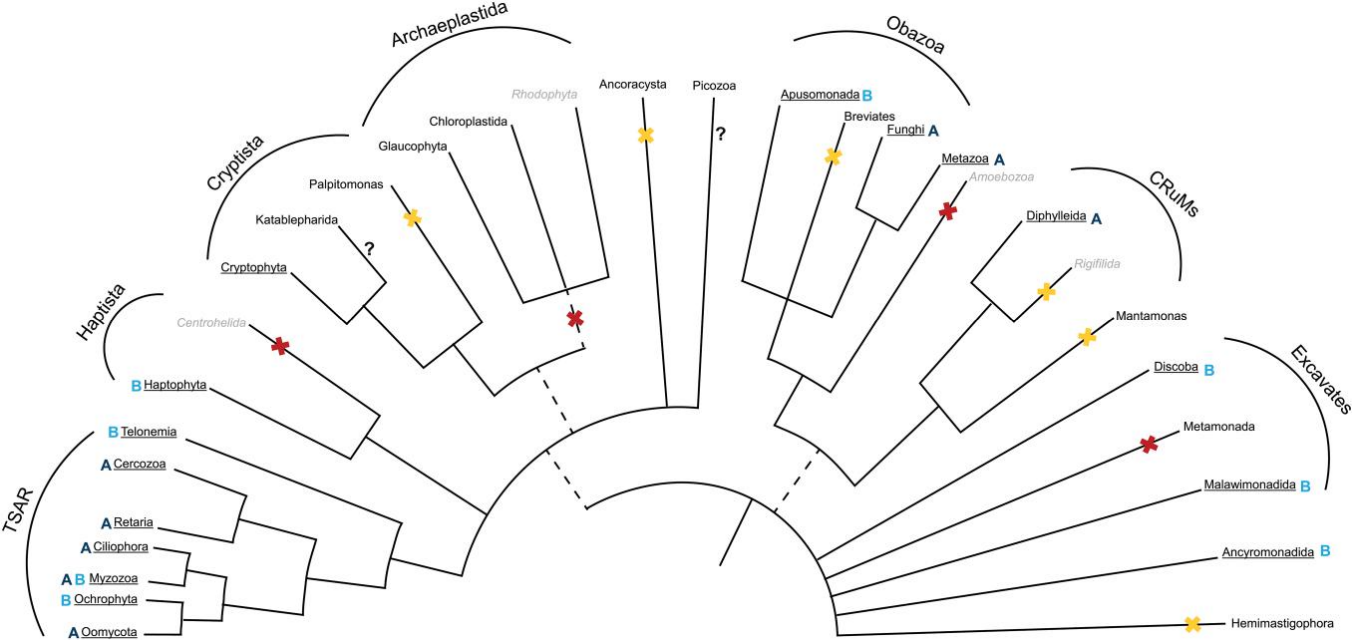
Presence and absence of the Adgb gene across eukaryotic evolution. Taxa with putative Adgb genes are underlined. The orthologous groupings provided by possvm are indicated by capital letters: homologous to ADGB a) and putative paralogs b). Adgb is absent in Centrohelida, Archaeplastida, and Metamonada lineages (red cross). Additional absences are possible in the lineages highlighted by yellow crosses; however, lineages were represented by only one to two genomes. The question marks indicate lineages that were not represented in the dataset. Lineages in gray and italics have lost the flagellum during the course of evolution (Derderian et al. 2023). Adapted and modified from Burki et al. 2020.

The lack of Adgb homologs in selected taxa from the fungi clade was initially interpreted as a restricted occurrence in metazoan taxa (Hoogewijs et al. 2012). The availability of more sequenced genomes from unicellular eukaryotes facilitated identifying many more putative Adgb homologs in a variety of flagellated eukaryotes. Adgb sequences are in fact widely distributed with occurrences in, among others, Fungi, Haptophyta, Alveolata, Amorphea, and Euglenozoa. Given the current topology of the eukaryotic tree (Al Jewari and Baldauf 2023), the Adgb gene would therefore have emerged before the split of Amorphea, Discoba, and Diaphoretics. A summary of ortholog distribution and Adgb-positive lineages is provided in Fig. 8.

Our phylogenetic analysis highlights both the power and limitations of orthology inference across billion-year timescales. The possvm algorithm identified two sequence groups that could represent either (i) paralogs arising from an ancient duplication in the common ancestor of TSAR and Opisthokonta (∼1.5 to 2 billion years ago) or (ii) orthologs whose extreme sequence divergence confounds accurate phylogenetic placement. Multiple lines of evidence favor the orthology interpretation: the bootstrap support of the node separating these two groups is very weak, and no lineage analyzed has retained both paralogous copies. The paralogous group contains several lineages that are known for extreme sequence divergence. Dinophyceae have undergone several rounds of whole-genome duplications (Lin 2024), resulting in highly derived genomes with exceptionally long branch lengths. The parasitic Perkinsozoa (Itoïz et al. 2021) show the elevated evolutionary rates characteristic of host-adapted parasites. These changes cannot be sufficiently resolved in a single gene tree and might mask true homology of all Adgb sequences analyzed. Even if the sequences were true paralogs, they still seem to have retained their original function. In both groups, the gene occurs only as a single copy exclusively in flagellated taxa and has been lost in closely related nonflagellated lineages, indicating no change in the selective constraints.

Of note, the Adgb gene appears to have been lost in several nonflagellated clades such as higher Fungi, Amebae, Centrohelida, and Apicomplexa and is also missing from model organisms lacking motile cilia, namely *D. melanogaster* and *C. elegans* and their associated relatives (Koay et al. 2021). This phylogenetic pattern strongly suggests a tight functional coupling of Adgb to the presence of flagella. At the same time, duplication events of Adgb are rare, and after well-described whole-genome duplications, such as in vertebrate evolution, divergence of paramecia, and the dinoflagellate lineage (McGrath et al. 2014; Lin 2024), additional copies of the Adgb locus are quickly lost. The considerable evolutionary stability likely reflects Adgb's integration into the core ciliary machinery as a nonredundant component. Gene dosage appears tightly constrained, with no tolerance for either duplication (which could disrupt stoichiometry in ciliary complexes) or loss (which abolishes function). This stringent constraint has enforced rigid single-copy maintenance across the eukaryotic tree, making Adgb a rare example of a globin lineage where evolutionary innovation occurred not through duplication and diversification, but through structural modification (domain permutation and fusion) of a single, irreplaceable ancestral gene. Our observations also point to an important contrast with other “non-classical” mammalian globins. Neuroglobin, although broadly distributed, displays lineage-specific losses, while Cytoglobin is similarly dispensable in certain clades (Hoffmann et al. 2011; Burmester and Hankeln 2014; Walt et al. 2026). Both proteins perform adaptive but replaceable roles in stress protection and redox homeostasis. Adgb, in contrast, is consistently maintained in lineages with motile cilia, indicating unusually strong evolutionary retention and positioning it as a stable element within an otherwise dynamic gene family.

Consistent with this deep evolutionary conservation, our experimental data provide independent support for an ancestral origin of Adgb as a core component of the ciliary gene repertoire. We show that the association of Adgb expression with ciliated cell types can be traced back to the most basal representatives of multicellular organisms. We demonstrate that Adgb is also expressed in ciliated cell types that are specific to certain phyla, such as choanocytes in sponges and comb cells of ctenophores, indicating that Adgb is an ancient member of the basal ciliary gene repertoire. In line with a regulatory factor X (RFX)-mediated regulation of mammalian Adgb transcription (Koay et al. 2021; Osterhof et al. 2026), Adgb expression in the choanoflagellate *S. rosetta* is regulated by the ancient ciliary transcription factor cRFXa. Although ciliary Adgb mRNA expression is detected across cnidarians, placozoa, ctenophorans, and poriferans, the number of Adgb-positive cell types is markedly higher in the cnidarian *N. vectensis* compared with other clades, and Adgb-positivity does not always coincide with ciliation. A similar pattern is observed in another anthozoan, the stony coral *Stylophora pistillata* (Fig. S7; Levy et al. 2021), but not in the hydrozoan *H. vulgaris* (Fig. 2c), hinting at a possible functional diversification of Adgb within anthozoans. Cnidarians therefore represent an interesting taxon for further studies of Adgb's function.

In functional terms, the divergence between unicellular and metazoan Adgb is striking. While ablation of Adgb in *T. thermophila* only modestly perturbs ciliary motility (Joachimiak et al. 2021), a knockout in mammals causes severe defects in sperm morphology and male infertility (Keppner et al. 2022). This discrepancy raises the possibility that the acquisition of the globin domain, the main structural innovation distinguishing metazoan Adgb from its unicellular homologs, conferred additional or specialized roles in flagellar motility and reproductive biology. The versatile nature of globins is well-established with functional links to O_2_ metabolism, nitric oxide signaling or redox regulation (Keppner et al. 2020). Integration of a globin domain into an ancestral ciliary protein scaffold may have endowed Adgb with additional O_2_- or redox-sensitive regulatory functions, enhancing the complexity of flagellar motility control and reproductive processes in multicellular organisms. Such a transition mirrors broader patterns in globin evolution, where domain fusion and diversification repeatedly facilitated functional innovation in specific cellular contexts. Notably, chimeric globin-coupled sensors and flavohemoglobins are abundant across prokaryotes (Vinogradov et al. 2007, 2013; Eduardo and Gilles-Gonzalez 2017), yet—with the exception of glb-33, a putative globin-coupled transmembrane receptor of unknown function in nematodes (Tilleman et al. 2015)—comparable globin-containing chimeras are essentially absent in metazoans, underscoring the exceptional nature of the Adgb architecture.

Collectively, our findings position Adgb at the intersection of globin biology, ciliary function, and the evolution of multicellularity. The retention of Adgb in flagellated lineages and its integration into conserved ciliogenic regulatory circuits underscore its ancient evolutionary role, while domain innovation and lineage-specific expression patterns highlight its potential for functional diversification. Adgb thus represents a paradigmatic case of how protein family members can be employed across deep evolutionary time to meet the demands of increasingly complex cellular and organismal contexts.

## Material and methods

### Animal husbandry and spawning induction

Adult *N. vectensis* were maintained at 17 °C in 12 parts per thousand (ppt) artificial seawater (ASW, InstantOcean) in a dark incubator. Animals were fed once weekly with freshly hatched *Artemia* nauplii. Tanks were cleaned, and the water was exchanged once per week. To induce spawning, animals were separated by sex into individual tanks, the water was replaced with fresh ASW, and both male and female cultures were exposed overnight to elevated temperature (25 °C) and bright light. Freshly released egg sacs from female tanks were fertilized by adding water from male tanks. Embryos were maintained at 21 °C until reaching the desired developmental stage.

Rotifers (*Brachionus plicatilis*) were cultured as food organisms. A starter culture was obtained commercially and maintained in 15 ppt ASW at room temperature. Rotifers were fed twice daily with RG Complete (Reed Mariculture, USA). Two-thirds of the culture volume were replaced twice per week, and the culture vessels were cleaned accordingly. Rotifers were harvested daily prior to feeding.

Adult *M. leidyi* were kept in shallow glass dishes at 21 °C in 30 ppt ASW under continuous bright light. Animals were fed daily with freshly hatched *Artemia* nauplii, and the water was exchanged three times per week. For induction of spawning, adults were conditioned for one week by daily feeding with rotifers, then transferred to darkness overnight. Spawning water was collected the following morning and screened for eggs and larvae, which were subsequently reared separately. Larvae were fed daily with rotifers for 10 d, followed by freshly hatched *Artemia* as they matured.

### Whole-mount in situ hybridization

In situ hybridization of Adgb mRNA was performed on whole *Nematostella* embryos and larvae via RNA scope (ACD Bio). Animals were fixed in 4% paraformaldehyde (PFA), dehydrated and processed as described before (Gross-Thebing 2020). Before the change of every reagent, the animals were collected at the bottom of the tube by gentle flicking. Probe hybridization was prolonged to 4 h at 40 °C under gentle agitation in a bacterial shaker (80 rpm), and animals were stored ON in 5× saline-sodium citrate (SSC). All washing steps were done three times (1× short, 2× 10 min). As fluorophore, TSA Vivid Dye 650 (1:750, ACD Bio) was used. A bacterial probe (ACD Bio) was used as negative control. Animals were counterstained with 4′,6-diamidino-2-phenylindole (DAPI) and mounted with Prolong Gold (Thermo Fisher) or processed further for immunostaining.

For *Mnemiopsis*, cydippid larvae (8 to 10 dph) were fixed overnight at 4 °C with 15% Rain-X in 30% ASW. After several washes in ASW, animals were postfixed for 1 h in 4% PFA in 30% ASW and then washed in 1× phosphate-buffered saline (PBS). Permeabilization was done for 2 h in PBS with 1% Triton-X. Samples were treated with 3% H_2_O_2_ for 10 min, washed twice in PBS with 0.5% Triton-X and equilibrated with probe diluent before starting the whole-mount RNA Scope protocol (customized kit, ACD Bio). Briefly, a probe against *Mnemiopsis* Adgb was diluted 1:50 in probe diluent and hybridized for 2 h at 40 °C under gentle agitation in a bacterial shaker (80 rpm). The signal was amplified with diluted Amp1 and Amp2 solutions and developed with horseradish peroxidase diluted in 5× SSC. As fluorophore, TSA Vivid Dye 650 (1:750, ACD Bio) was used. Image acquisition was done on a Stellaris 8 (Leica), and subsequent image editing was performed with Icy (Version 2.5.4.0).

### Immunofluorescence

Animals were fixed as described above, blocked in 5% normal goat serum in PBS and incubated over night with antiacetylated tubulin antibody (1:500, Santa Cruz). After several washing steps, animals were incubated with secondary antibody (Alexa Fluor 633 or 488, 1:250, Invitrogen), counterstained with DAPI and mounted in ProLong Gold (Thermo Fisher). Image acquisition was done on a Stellaris 8 (Leica), and subsequent image editing was performed with Icy (Version 2.5.4.0).

### Identification, characterization, and phylogenetic analysis of Adgb homologs

Adgb homologs in distant species were searched via reciprocal BLAST, starting from different known Adgb sequences. Both the EukProt TCS dataset and NCBI database were used for the analysis. Candidate sequences were length filtered (>800 aa) and collapsed at 98% identity level. Protein domains and similarities of selected sequences were predicted and annotated with InterProScan (https://www.ebi.ac.uk/interpro/ (File S6) (Paysan-Lafosse et al. 2023)). This length cutoff is equivalent to a PANTHER Androglobin domain-based candidate identification, since no sequence under 800 aa showed any hits from PANTHER Androglobin, whereas 98% where positive for the PANTHER Androglobin domain. Sequences were aligned with MAFFT v7 with the L-INS-i algorithm for accurate alignment of sequences with conserved domains. The alignment was trimmed using trimAl v 1.4.1. Subsequent maximum likelihood phylogenetic analysis was performed with IQTREE 3.0.1 (Wong et al. 2025). The best-fit substitution model (Q.INSECT + F + R7) was selected using ModelFinder based on the Bayesian Information Criterion. Branch support was assessed using 1000 ultrafast bootstrap replicates. Taxonomic information for all sequences was retrieved from the NCBI Taxonomy database using Entrez queries. Species were classified into major eukaryotic supergroups (Opisthokonta, Stramenopiles/Alveolates/Rhizarian, Excavata, Archaeplastida, Amoebozoa, and others) following current eukaryotic taxonomy.

### Orthology inference

Orthogroup assignment was performed using possvm v1.2 (Grau-Bové and Sebé-Pedrós 2021), which implements a species overlap algorithm combined with Markov Cluster algorithm clustering. The unrooted gene tree was processed with iterative midpoint rooting (ten iterations) to identify optimal root placement. Human ADGB (NP_078970.3) was designated as the reference gene for orthogroup naming. Sequences were assigned species prefixes following possvm requirements. The analysis identified orthogroups based on speciation events inferred from the species overlap algorithm, with orthogroup support values representing the ultrafast bootstrap value at the most recent common ancestor node of each group.

### Protein structure prediction with AlphaFold2

Protein 3D structures were predicted using AlphaFold v2.1.2 (Jumper et al. 2021), installed via the high performance computing module system on the MOGON II cluster (Johannes Gutenberg University Mainz). All predictions were run on NVIDIA GPUs (CUDA 11.3.1, one GPU per job) with 24 GB RAM and two central processing units allocated per task. Input protein sequences were provided in FASTA format. For sequences exceeding the maximum supported input length, the proteins were split into two nonoverlapping parts at positions avoiding disruption of annotated or predicted domains. Each fragment was folded separately, and results for split proteins are reported for both parts.

AlphaFold was executed with the monomer model preset and full_dbs database preset, using the following reference databases: UniRef90 (release 2021_03), UniRef30/UniClust30 (release 2021_03), MGnify (release 2022_05), PDB70 (as of May 2020), and the Protein Data Bank (PDB/mmCIF, max template date set to May 14, 2020). Multiple sequence alignments were automatically generated by AlphaFold against these databases. Template information was drawn from PDB70 and PDB/mmCIF files where available. All predictions were performed with Amber relaxation enabled (−use_gpu_relax flag). For each sequence or fragment, five models were generated and ranked according to the AlphaFold internal scoring metric.

The complete set of raw prediction outputs, including input FASTA sequences, multiple sequence alignments, model ranking JSON files, unrelaxed and relaxed structures, and result pickles, is publicly available via Zenodo under accession 10.5281/zenodo.17194860.

### Data acquisition

We employed publicly available transcriptomic data from previously published studies. Raw data for RNA-seq experiments were downloaded from the EBI (https://www.ebi.ac.uk/ena/browser/) or NCBI (https://www.ncbi.nlm.nih.gov/sra) web servers in fastq format. Read count matrices of scRNA-seq data were also downloaded from these repositories or taken from the Supplementary material of the corresponding publication. The full list of datasets can be found in File S7.

### Processing of bulk RNA-sequencing data

Quality scores of RNA-seq data were evaluated using fastqc (https://www.bioinformatics.babraham.ac.uk/projects/fastqc/). Adapter trimming and quality filtering were performed using bbduk from the BBtools suite (https://sourceforge.net/projects/bbmap/). Parameters were chosen depending on the sequence length and overall quality of the dataset, and selected mode for trimming was retained for all datasets of the same study. Reads were then mapped against the corresponding reference genome (Table S1) with HISAT2 (Kim et al. 2019) and expression levels quantified with StringTie as transcripts per million (Pertea et al. 2015). For the developmental time course of *A. queenslandica* embryos, the raw data were mapped, and the resulting count data were imported in R. Whole embryo transcriptomes were then ordered using the pseudotime function of Monocle 3 (Trapnell et al. 2014).

### Differential gene expression and ontology analysis

Differential gene expression analysis was performed in R using Bioconductor package DESeq2 (Love et al. 2014). Gene ontology enrichment analysis was subsequently performed with g:Profiler (Raudvere et al. 2019) for *Amphimedon* and *Mnemiopsis*. For *Nematostella*, transcripts were functionally annotated with eggNOG-mapper (Cantalapiedra et al. 2021), and gene ontology term enrichment was performed with topGO (Alexa and Rahnenfuhrer 2023).

### Processing of scRNA-seq data

Single-cell sequencing data were downloaded as count matrices from the EBI or NCBI web server. Quality filtering and cluster analysis were performed in R with Seurat 4 (Hao et al. 2021). Cell identities were either kept if metadata were available, or were assigned via expressed marker genes, in accordance with the original publications. Differential expression analysis was performed using Seurat's build-in “find.markers” function with default parameters. Pearson correlation coefficients were calculated from expression matrices. Gene ontology enrichment on differentially expressed genes was performed using g:Profiler or topGO. A subset of datasets or single clusters were subjected to pseudotime and trajectory analysis using Monocle 3.

## Supplementary Material

msag147_Supplementary_Data

## Data Availability

The data underlying this article are available in the article and in its online Supplementary material.
