# Supplementary material for: Ancient conservation of androglobin expression reveals its evolutionary link to ciliary processes
Source: Mol Biol Evol. 2026 Jun 12;43(7):msag147. doi: 10.1093/molbev/msag147 (PMC13331133; doi:10.1093/molbev/msag147)
Supplement: msag147_Supplementary_Data [file msag147_supplementary_data.zip › Osterhof et al Supplemental.docx]

**Supplemental files**

**Ancient conservation of androglobin expression reveals its evolutionary link to ciliary processes**

Carina Osterhof^1,2^, David Teschner^3^, Michelle Balling^1^, Antonia Herwig^2^, Charlotte Duda^2^, Gaëlle Botton-Amiot^4^, Andreas Hildebrandt^3^, Simon Sprecher^4^, Thomas Hankeln^1+^ and David Hoogewijs*^2+^

^1^Institute of Organismic and Molecular Evolution, Molecular Genetics and Genome Analysis, Johannes Gutenberg University, Mainz, Germany

^2^Department of Endocrinology, Metabolism and Cardiovascular System, University of Fribourg, Fribourg, Switzerland

^3^Institute of Computer Science, Johannes Gutenberg University, Mainz, Germany

^4^Department of Biology, Institute of Zoology, University of Fribourg, Fribourg, Switzerland

^+^These authors contributed equally to this work.

*correspondence: [david.hoogewijs@unifr.ch](mailto:david.hoogewijs@unifr.ch)

**Supplementary table 1:** List of organisms, their corresponding reference genomes and annotation versions used in bulk RNA-seq analysis in this study.

| Organism | Genome version | annotation |
| --- | --- | --- |
| *Amphimedon queenslandica* | Aqu1 (GCA_000090795.1) | Aqu1.52 (gene set v2.1) |
| *Mnemiopsis leidyi* | MneLei_Aug2011 (GCA_000226015.1) | MneLei_Aug2011.52 |
| *Nematostella vectensis* | Nvec200  https://simrbase.stowers.org/ | Nvec2.gtf  https://simrbase.stowers.org/ |
| *Salpingoeca rosetta* | Proterospongia_sp_ATCC50818  (GCA_000188695) | Proterospongia_sp_ATCC50818.57.gtf |

**Supplemental Figures**

**Supplementary figure 1:** Maximum likelihood reconstitution of Adgb phylogeny across eukaryotes. Major clades are labelled on the left and selected phyla on the right. Conserved domains in the sequences were annotated with InterProScan, green indicating presence and orange absence. The column “ADGB ortholog” refers to the clustering of sequences performed by the possvm algorithm, that predicts two paralogous groups of Adgb sequences distributed over a wide variety of taxa.

**Supplementary figure 2:** Adgb expression during development of *Nematostella* larvae. Adgb mRNA expression is higher in the aboral part carrying the ciliated organ in both early and late-stage planula. Data from: (Gilbert et al., 2022)

**Supplementary figure 3:** A) In bulk mRNA sequencing data derived from sorted *Amphimedon queenslandica* (Aqu) cell populations, Adgb mRNA expression is highest in choanocytes, but shows prominent inter-individual differences. B) Adgb expression during development of Aqu. Pseudo-time sorted transcriptomes from single embryos during development of Aqu. Adgb expression is highest during mid-embryogenesis and correlates strongly with ciliary marker rsh protein 3. Emb – embryogenesis; cl – competent larvae; pl – post-competent larvae.

**Supplementary figure 4:** FoxJ1 and Adgb mRNA expression in different clusters of *Mnemiopsis leidyi* scRNA-Seq data (ref. Figure 4B). The two Adgb expressing clusters (“Comb cells” and “Unknown”) also display an enrichment of FoxJ1-positive cells.

**Supplementary figure 5:** GO-terms correlated with Adgb mRNA expression in 4 different species of Placozoa. From scRNA-seq count matrices, we computed correlation coefficients between Adgb and all other genes, and performed GO enrichment analysis on the top 100 genes. Here, the top 20 GO terms (sorted by FDR) from the biological process category are shown. A) *Hoilungia hongkongensis*; B) *Cladtertia collaboinventa*; C) *Trichoplax adhaerens*; D) *Trichoplax sp. H2.*

**Supplementary figure 6:** Predicted structure of calpain 7 of the fish *Danio rerio.* Arrows point at the characteristic beta-sheet structures, which can also be found in the structure of Adgb (ref. Figure 6).

**Supplementary figure 7:** Adgb mRNA expression in the stony coral *Stylophora pistillata*. Adgb positivity is widely distributed, with a few hotspots in meta cell clusters “mitotic host cell”, several neuronal clusters and three metacells labelled as “unknown”.

**
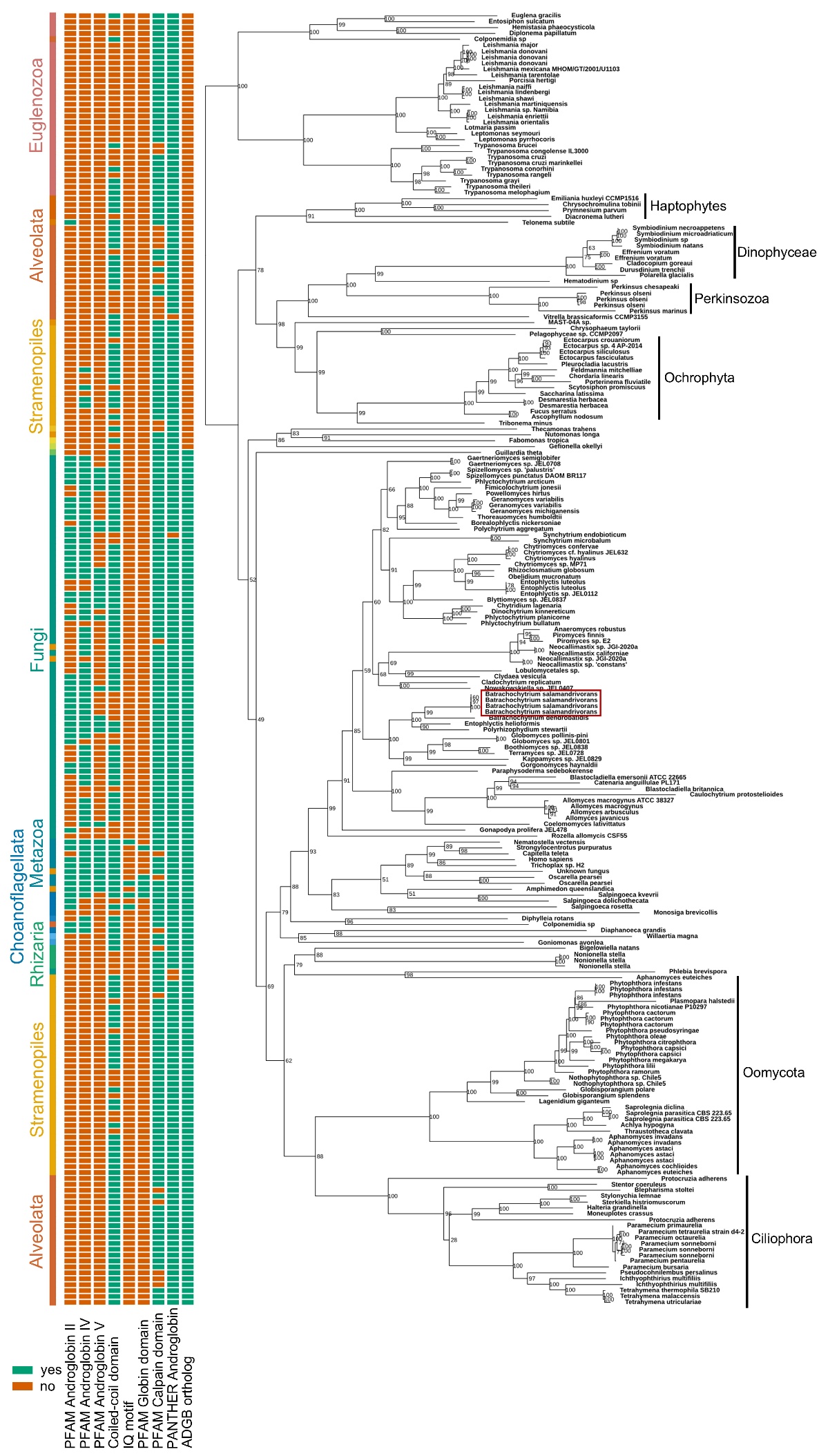
**

**Supplementary figure 1:** Maximum likelihood reconstitution of Adgb phylogeny across eukaryotes. Major clades are labelled on the left and selected phyla on the right. Conserved domains in the sequences were annotated with InterProScan, green indicating presence and orange absence. The column “ADGB ortholog” refers to the clustering of sequences performed by the possvm algorithm, that predicts two paralogous groups of Adgb sequences distributed over a wide variety of taxa.


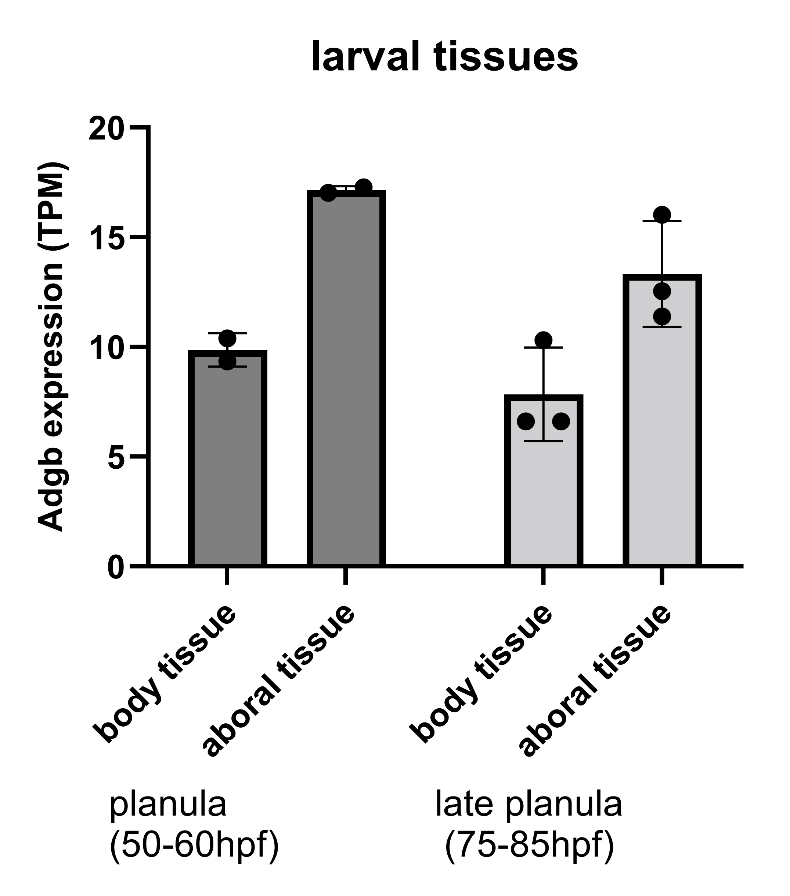


**Supplementary figure 2:** Adgb expression during development of *Nematostella* larvae. Adgb mRNA expression is higher in the aboral part carrying the ciliated organ in both early and late-stage planula. Data from: (Gilbert et al., 2022)


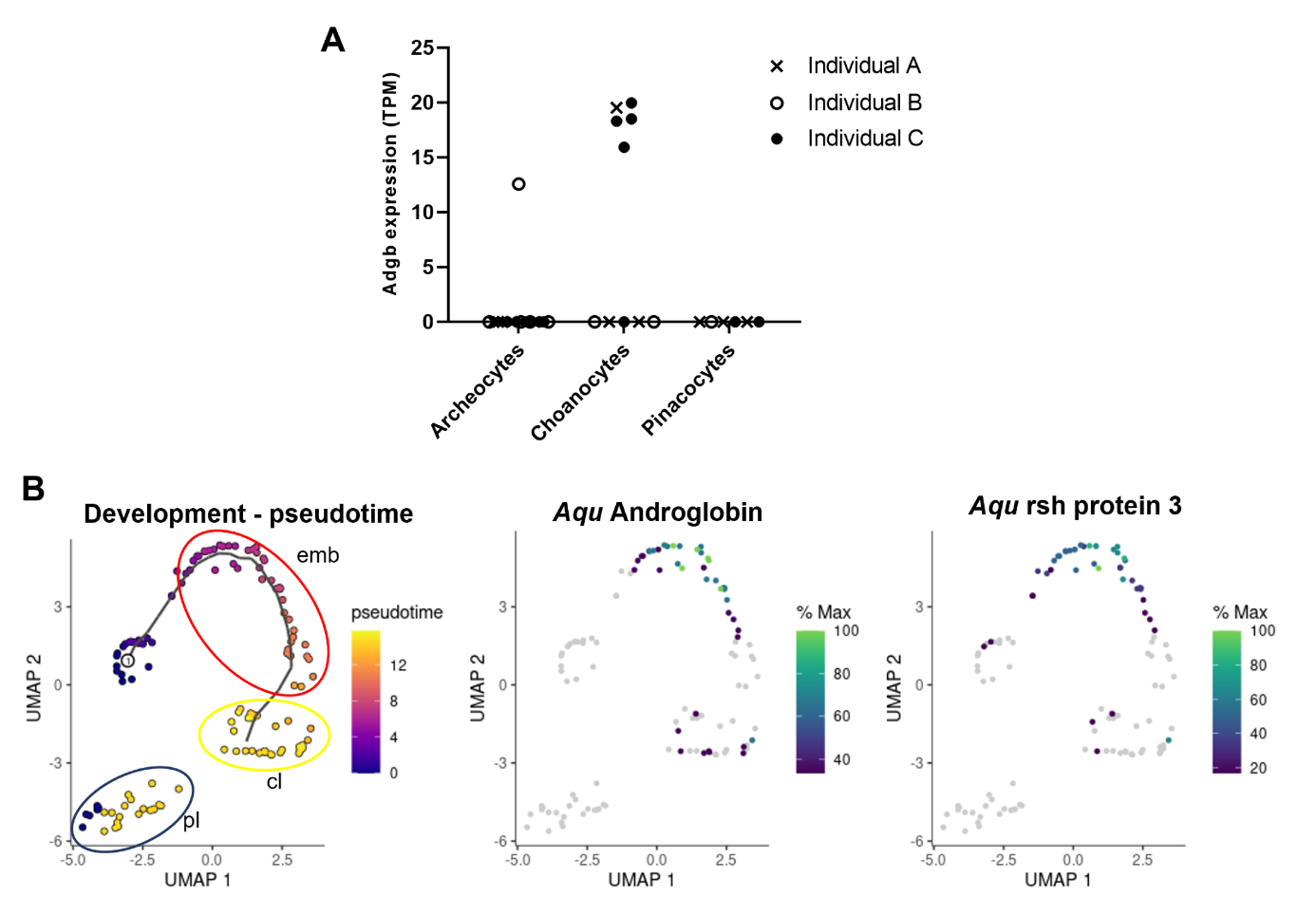


**Supplementary figure 3:** A) In bulk mRNA sequencing data derived from sorted *Amphimedon queenslandica* (Aqu) cell populations, Adgb mRNA expression is highest in choanocytes, but shows prominent inter-individual differences. B) Adgb expression during development of Aqu. Pseudo-time sorted transcriptomes from single embryos during development of Aqu. Adgb expression is highest during mid-embryogenesis and correlates strongly with ciliary marker rsh protein 3. Emb – embryogenesis; cl – competent larvae; pl – post-competent larvae.


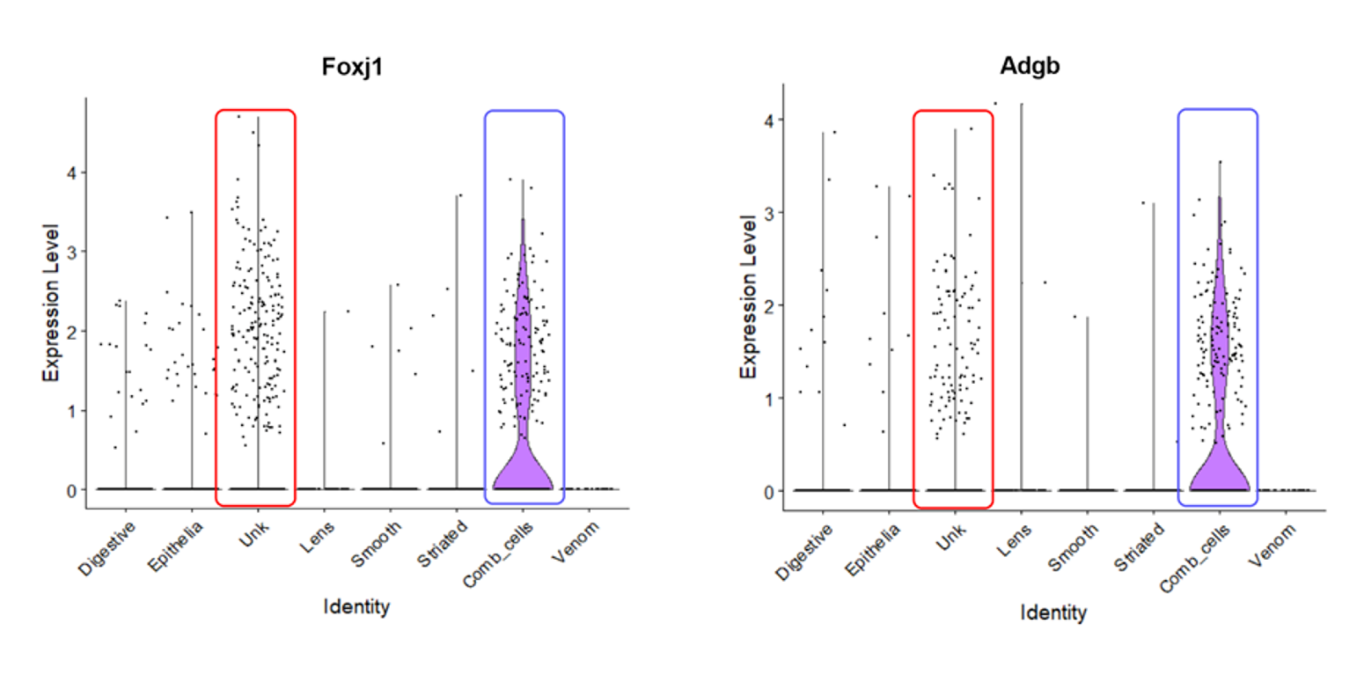


**Supplementary figure 4:** FoxJ1 and Adgb mRNA expression in different clusters of *Mnemiopsis leidyi* scRNA-Seq data (ref. Figure 4B). The two Adgb expressing clusters (“Comb cells” and “Unknown”) also display an enrichment of FoxJ1-positive cells.


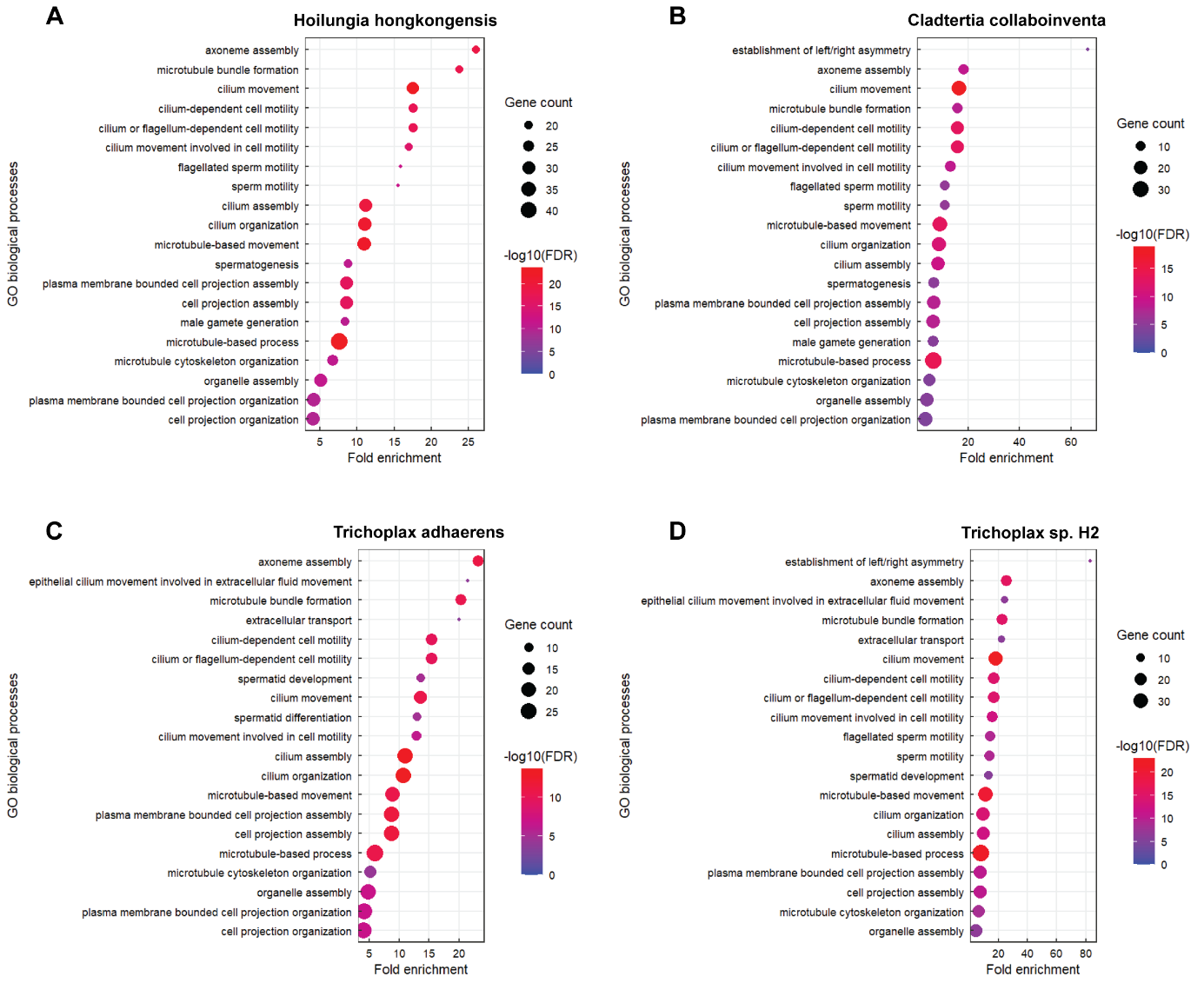


**Supplementary figure 5:** GO-terms correlated with Adgb mRNA expression in 4 different species of Placozoa. From scRNA-seq count matrices, we computed correlation coefficients between Adgb and all other genes, and performed GO enrichment analysis on the top 100 genes. Here, the top 20 GO terms (sorted by FDR) from the biological process category are shown. A) *Hoilungia hongkongensis*; B) *Cladtertia collaboinventa*; C) *Trichoplax adhaerens*; D) *Trichoplax sp. H2.*


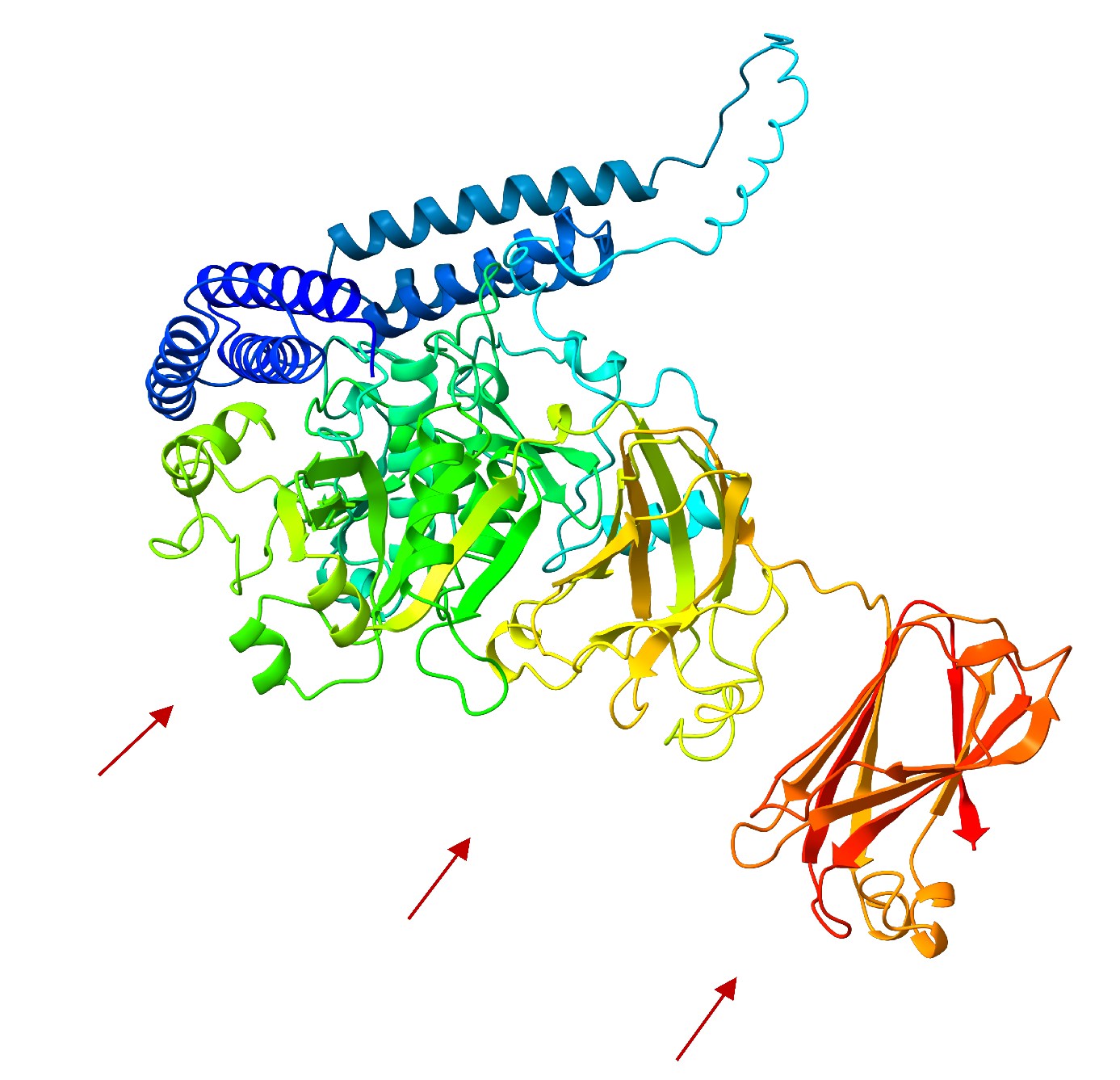


**Supplementary figure 6:** Predicted structure of calpain 7 of the fish *Danio rerio.* Arrows point at the characteristic beta-sheet structures, which can also be found in the structure of Adgb (ref. Figure 6).


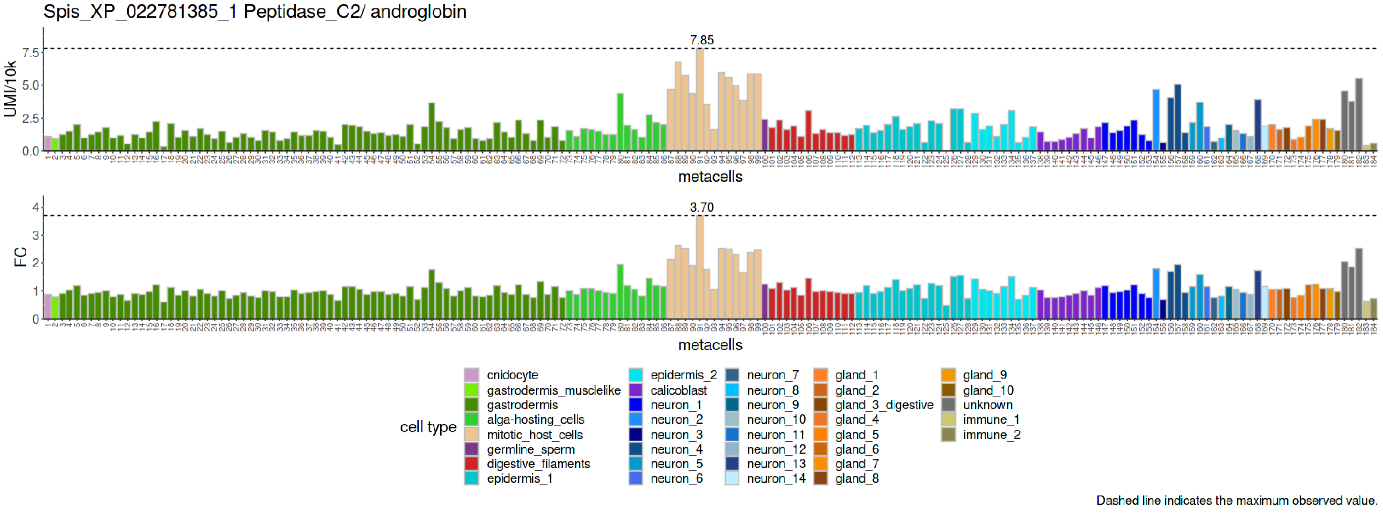


**Supplementary figure 7:** Adgb mRNA expression in the stony coral *Stylophora pistillata*. Adgb positivity is widely distributed, with a few hotspots in meta cell clusters “mitotic host cell”, several neuronal clusters and three metacells labelled as “unknown”.

**Supplementary files:**

Supplementary file 1 Condensed Protein domains and similarities based on InterProScan

Supplementary file 2 Nvec_gastrointestinal_Adgb_correlated_genes

Supplementary file 3 Aqu_collagen ciliated_Adgb_correlated_genes

Supplementary file 4 Mley_unknown cluster_Adgb_correlated_genes

Supplementary file 5 Placozoa_Adgb_correlated_genes

Supplementary file 6 Protein domains and similarities based on InterProScan raw output

Supplementary file 7 Public_datasets_accession
